# Determination and Prediction of Amino Acid Digestibility in Rapeseed Cake for Growing-Finishing Pigs

**DOI:** 10.3390/ani14192764

**Published:** 2024-09-25

**Authors:** Hui Tang, Ganyi Feng, Jingfeng Zhao, Qing Ouyang, Xiaojie Liu, Xianji Jiang, Menglong Deng, Zhengjun Xie, Fengming Chen, Xihong Zhou, Rui Li, Yulong Yin

**Affiliations:** 1 Hunan Co-Innovation Center of Animal Production Safety, College of Animal Science and Technology, Hunan Agricultural University, Changsha 410128, China; tanghui9580@163.com (H.T.); zhaojf@stu.hunau.edu.cn (J.Z.); qingouyang413@163.com (Q.O.); liuxiaojiefree@163.com (X.L.); jxj8888888888@163.com (X.J.); maxdml666@gmail.com (M.D.); yinyulong@isa.ac.cn (Y.Y.); 2 Key Laboratory of Agro-Ecological Processes in Subtropical Region, Institute of Subtropical Agriculture, Chinese Academy of Sciences, Changsha 410125, China; yanergougoujun@gmail.com (G.F.); xhzhou@isa.ac.cn (X.Z.); 3 Twins Group Co., Ltd., Nanchang 330096, China; xiezj@sbtjt.com; 4 Hunan Provincial Key Laboratory of the TCM Agricultural Biogenomics, Changsha Medical University, Changsha 410129, China; cfming@stu.hunau.edu.cn

**Keywords:** rapeseed cake, amino acids digestibility, prediction model

## Abstract

**Simple Summary:**

Nutritional composition of rapeseed cake varies considerably due to differences in cultivars, growing conditions, harvesting time, and processing techniques, making it difficult to formulate precise diets. Therefore, we conducted a study to determine the chemical composition and standardized ileal digestibility (SID) of the amino acids (AA) in rapeseed cake and to test the hypothesis that their nutritional value is influenced by their origin. At the same time, we attempted to establish a predictive equation for SID based on the chemical composition of AA in RSC fed to pigs.

**Abstract:**

Objective: The experiment was conducted to determine the apparent or standardized ileal digestibility (AID or SID) of crude protein (CP) and amino acids (AA) in 10 rapeseed cake samples fed to pigs, and to construct predictive models for the SID of CP and AA based on the chemical composition of rapeseed cakes. Methods: Twenty-two cannulated pigs (initial body weight: 39.8 ± 1.2 kg) were assigned to two 11 × 3 incomplete Latin square designs, including an N-free diet and 10 diets containing rapeseed cake. Each experimental period included 5 days of adaptation and 2 days of ileal digesta collection. Titanium dioxide (TiO_2_) was added at 0.3% to all the diets as an indigestible marker for calculating the ileal CP and AA digestibility. Results: The coefficients of variation (CV) of the content of crude fat (EE), crude fiber (CF), neutral detergent fiber (NDF), acid detergent fiber (ADF), and total glucosinolates (TGS) in 10 samples of rapeseed cake were greater than 10%. The standardized ileal digestibility (SID) of crude protein (CP), lysine (Lys), methionine (Met), threonine (Thr), and tryptophan (Trp) in rapeseed cake was 73.34% (61.49 to 81.12%), 63.01% (41.41 to 73.10%), 69.47% (50.55 to 88.16%), 79.61% (74.41 to 87.58%), and 94.43% (91.34 to 97.20%), respectively. The best prediction equations for SID_CP_, SID_Lys_, and SID_Val_ were as follows: SID_CP_ = 90.124 − 0.54NDF (R^2^ = 0.58), SID_Lys_ = 100.107 − 1.229NDF (R^2^ = 0.94), and SID_Val_ = 151.012 − 2.990TGS (R^2^ = 0.57). Conclusion: Overall, great variation exists among the 10 rapeseed cakes, and the NDF, TGS, and heating temperature can be used as the key predictors for the SID of CP and AA.

## 1. Introduction

The demand for protein feed resources is expected to rise due to global population growth and the increasing need for animal-based food [1,2]. Data from the National Bureau of Statistics show that animal production in China is highly dependent on the imported soybean, which significantly affects national food security. The Ministry of Agriculture and Rural Affairs of China has drawn up several strategies to reduce the utilization of soybean meal in animal feed. Rapeseed is one of the major global sources of vegetable oil and protein feed, ranking second only to soybeans in trade volume [2]. Rapeseed cake (RSC), a by-product after oil extraction, contains around 36% of crude protein (CP) but offers an unbalanced amino acid profile [2,3,4,5,6,7]. Rapeseed cake has lower lysine (Lys) contents than soybean meal, and it is rich in sulfur-containing amino acids and more cost-effective [2,3,4,5,6,7]. Studies have shown that rapeseed cake can effectively replace soybean meal in the diets of adult pigs [5,7,8]. However, greater variations in the nutrient composition of RSC exist due to the differences in cultivars, growing conditions, harvest time, and processing technology, making precision formulation difficult [2]. Accurate estimation of amino acid availability in diets is essential for low-protein diet systems [9], and the standardized ileal digestibility (SID) of animo acids is the gold standard for characterizing the amino acid availability in feed ingredients [9,10]. 

Therefore, we conducted research to determine the chemical composition and standardized ileal digestibility (SID) of amino acids (AA) in the RSC, and tested the hypothesis that its nutritional value is affected by its origin. Meanwhile, we tried to establish predicted equations for the SID of AA in the RSC fed to pigs based on its chemical composition.

## 2. Materials and Methods

### 2.1. Sources of Rapeseed Cake Samples

Ten RSC samples were selected from Guizhou (*n* = 1), Sichuan (*n* = 1), Anhui (*n* = 1), Shaanxi (*n* = 1), Henan (*n* = 1), Hubei (*n* = 2), and Hunan (*n* = 2) provinces and from Chongqing municipality (*n* = 1) (Table 1). The main production areas of rapeseed in China are in the Yangtze River Basin and its surrounding areas [11]. In our research, the selection of rapeseed cake samples was mainly concentrated in the Yangtze River Basin of China, but there was also a sample RSC5 from the Huaihe River Basin. The RSC ingredients were ground in a feed mill and used to prepare experimental diets. The samples were sieved through a 40-mesh screen, obtained using stratified sampling and the quadrant method, and stored at −20 °C until use [12]. 

### 2.2. Animals, Diets, and Experimental Design

A total of 22 castrated pigs [Duroc × (Yorkshire × Landrace), initial BW: (39.8 ± 1.7) kg], with a distal ileum T-cannula were assigned to two incomplete 11 × 3 Latin square designs and fed with 10 different diets containing RSC as the sole nitrogen source and one nitrogen-free diet for determining the basal endogenous losses of CP and AA (Table 2) [3,12,13]. All diets contained 0.30% of titanium dioxide (TiO_2_) as an indigestible marker for calculating the digestibility of CP and AA. Adequate amounts of vitamins and minerals were added to all diets according to the NRC (2012) [6] recommendations. The experiment consisted of three consecutive periods. Every experimental cycle included 5 days of adaptation followed by 2 days of ileal digesta collection. Six replicate samples were obtained for each diet treatment. All pigs were individually housed in metabolic cages (1.4 m × 0.7 m × 0.5 m) and maintained at a temperature of (23 ± 1) °C. Before each experimental cycle, the pigs were individually weighed, fed equal amounts at 0800 and 1700 h, totaling 4% of the average initial body weight, and had free access to water. 

### 2.3. Sample Collection and Preparation

During the 2 days of ileal digesta collection, ileal digesta was collected for 8 h daily from 8:00 a.m. to 4:00 p.m. each day. The procedure for collecting ileal digesta was as follows: the plug cap and inner sleeve were removed, and a plastic bag was attached to the T-shaped cannula port and secured with a rubber band to collect the ileal digesta. After collecting for a maximum of 30 min or obtaining approximately more than one-third of the volume of the plastic bag, the plastic bag was removed and immediately frozen at −20 °C to minimize bacterial fermentation [9,12]. At the end of each cycle, the frozen ileal digesta samples were combined and placed in a Vacuum-Freeze Dryer (SCIENTZ-50F/A, Ningbo Xinzhi Lyophilization Equipment Co., Ltd., Ningbo, China) for drying. After drying, the resulting samples were finely ground to pass through a 1 mm sieve.

### 2.4. Sample Analysis and Calculation

The levels of dry matter (DM), ether extract (EE), crude protein (CP), ash, calcium (Ca), and total phosphorus (TP) in the RSC were measured following AOAC (2006) [14] procedures [(DM,930.15), (EE, 920.39), (CP, 984.13), (ash, 942.05), (Ca, 968.08), (TP, 964.06)]. The amounts of crude fiber (CF), neutral detergent fiber (NDF), and acid detergent fiber (ADF) were determined using a fiber analyzer (ANKOM A200i Fiber Analyzer, Beijing ANKOM Technology Co., Ltd., Beijing, China) with fiber bags, as per the method outlined by Van Soest et al. (1991) [15]. The content of total glucosinolates (TGS) was analyzed using an Elisa kit (Fankew, Shanghai Kexing Trading Co., Ltd., Shanghai, China). The gross energy (GE) content of the RSC was determined using an automatic oxygen bomb calorimeter (HXR-6000 calorimeter, Hunan Huaxing Energy Instrument Co. Ltd., Changsha, China). 

We used the same method to detect the CP levels in the feed and in the freeze-dried ileal digesta samples as we did for the RSC. The analysis of the AA content in the rapeseed cake, feed, and ileal digesta samples followed these steps: First, we conducted acid hydrolysis using 6 M HCl, then measured the content of 15 amino acids using high-performance liquid chromatography (Agilent 1200, Agilent Technologies, Santa Clara, CA, USA). Methionine (Met) and cysteine levels were determined through the oxidative hydrolysis (method 982.30 E(a); AOAC, 2006) [14]. Tryptophan (Trp) was hydrolyzed with 10% KOH at 40 °C for 16–18 h, and its content was determined using spectrophotometry according to GB/T 15400-2018 [16]. The content of TiO_2_ in the diets and ileal digesta was analyzed using an inductively coupled plasma optical emission spectrometer (ICP-OES 5110, Agilent Technologies) according to the method of GB/T 5009.246-2016 [17]. The apparent ileal digestibility (AID) and SID of amino acids (%), in the RSC samples, were determined based on the method described by Stein et al. (2007) [10] using the following equation:

AID = [1 − (AA_d_ × T_r_)/(AA_r_ × T_d_)] × 100%

AA_d_ and T_d_ represent the concentrations of AA and TiO_2_ in the ileal digesta (g/kg of DM), respectively, while AA_r_ and T_r_ are the concentrations of AA and TiO_2_ in the RSC diets (g/kg of DM), respectively. The same equation was used to calculate the AID of CP:

IAA_end_ = [AA_d_ × (T_r_/T_d_)]

In the equation, IAA_end_ represents the basal endogenous loss of each AA (g/kg of DM intake, DMI), and AA_d_ and T_d_ represent the concentrations of AA and TiO_2_ in the ileal digesta from the growing-finishing pigs fed the N-free diet, respectively. The T_r_ represents the concentration of TiO_2_ in the N-free diet. The same equation was used to calculate the endogenous loss of CP:

SID = [AID + (IAA_end_/AA_d_) × 100%]

### 2.5. Statistical Analysis

We used SPSS 27.0 (SPSS Inc., Chicago, IL, USA) to assess the normality and equal variance of the data, and Z-scores were analyzed to identify outliers. The CORR procedure was employed to examine the correlation coefficients among the chemical composition and the AA digestibility (AID and SID of Lys, Met, Trp, and Thr) of the RSC samples. Stepwise regression was used to establish prediction equations for the SID of Lys, Met, Trp, and Thr of the RSC samples based on their chemical compositions. The best-fit equations were selected based on their relative standard deviation (RSD), R^2^, and *p*-value. *p* < 0.05 means significant difference and *p* < 0.01 means extremely significant difference; when R^2^ is closer to 1 and *p*-value represents a significant difference, the equation is considered more accurate.

## 3. Results

### 3.1. Chemical Composition and AA Profile of Rapeseed Cake and Its Diet

On the air-dry basis, the mean contents of GE, DM, CP, EE, ash, CF, NDF, ADF, Ca, TP, and TGS of the 10 RSC samples were 19.25 MJ/kg (18.45 to 20.84 MJ/kg), 91.62% (87.95% to 94.68%), 39.15% (35.15% to 43.61%), 8.11% (5.48% to 11.76%), 6.77% (5.99% to 7.75%), 9.34% (8.26% to 11.32%), 30.19% (21.89% to 48.61%), 17.68% (13.13% to 22.87%), 0.58% (0.50% to 0.62%), 1.16% (0.92% to 1.30%), and 22.35 μmol/g (20.25 to 25.02 μmol/g), respectively. The coefficients of variation (CV) of EE, CF, NDF, ADF, and TGS in the RSC samples were greater than 10% (Table 3). 

Except for leucine, the CV of other amino acids exceeded 10%. The concentrations of Lys, Met, Thr, and Trp in the 10 RSC samples were 1.80% (1.22% to 2.20%), 0.82% (0.71% to 1.29%), 1.61% (1.20% to 1.97%), and 0.31% (0.25% to 0.37%), respectively. 

The chemical composition and AA profile of rapeseed cake diet are shown in Table 4.

### 3.2. AID and SID of CP and AA

The AID values for CP, Lys, Met, Thr, and Trp in the 10 RSC samples were 59.72% (46.54% to 70.29%), 61.97% (39.94% to 72.23%), 65.31% (46.08% to 83.92%), 67.88% (62.81% to 75.86%), and 79.91% (73.45% to 84.12%), respectively (Table 5). Significant differences (*p* < 0.01) were observed in the AID values for CP, Lys, Met, and Trp.

The SID values of CP, Lys, Met, Thr, and Trp in the 10 RSC samples were 73.81% (61.92% to 81.19%), 63.02% (41.45% to 73.11%), 69.51% (50.56% to 88.28%), 79.75% (74.42% to 87.66%), and 94.63% (91.41% to 97.76%), respectively (Table 6 and Table 7). Significant differences (*p* < 0.01) were observed in the SID of CP, Lys, and Met.

### 3.3. Correlation Analysis and Prediction Equations for SID of CP and AA

**Figure 1:**
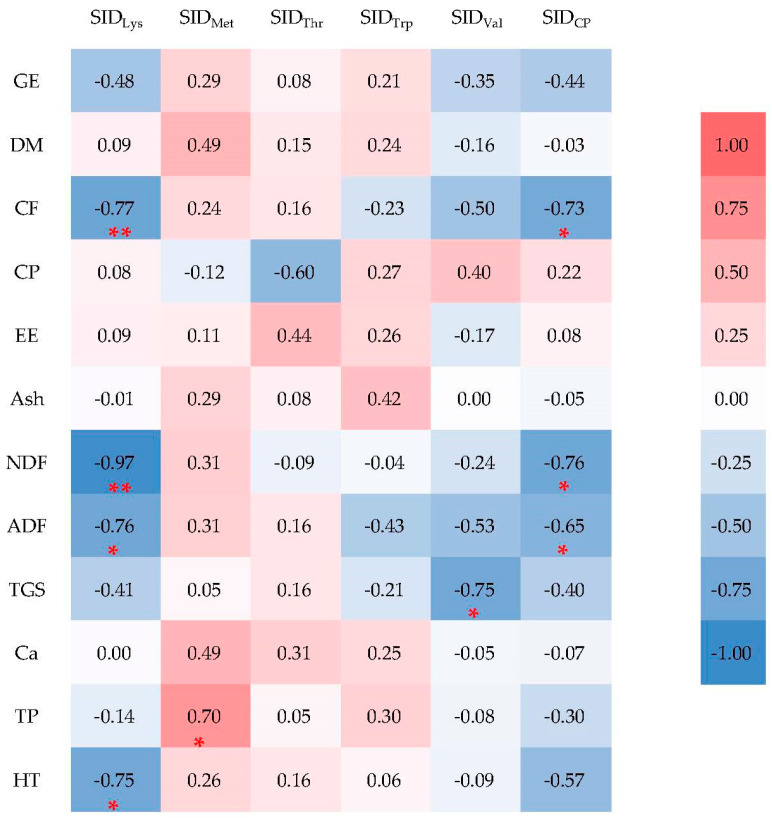
Correlation coefficients (r) among physical characteristics, chemical constituents, and the SID of the first four limiting amino acids of the 10 rapeseed cake samples. * means significant difference (*p* < 0.05); ** means extremely significant difference (*p* < 0.01). SID_CP_, SID_Lys_, SID_Met_, SID_Thr_, SID_Trp_, and SID_Val_ indicate the SID of CP, Lys, Met, Thr, Trp, and Val, respectively. DM, dry matter; GE, gross energy; CP, crude protein; EE, ether extract; Ash, crude ash; CF, crude fiber; NDF, neutral detergent fiber; ADF, acid detergent fiber; Ca, calcium; TP, total phosphorus; TGS, total glucosinolate; HT, heating temperature.

Significant negative correlation was found between the SID of Lys and heating temperature (*p* < 0.05, Figure 1), as well as with the levels of CF and NDF (*p* < 0.01, Figure 1). In Table 8, the optimal fitting equations for SID_CP_, SID_Lys_, and SID_Val_ are as follows: SID_CP_ = 90.124 − 0.540NDF (R^2^ = 0.58, RSD = 4.39, *p* < 0.05), SIDLys = 100.107 − 1.229NDF (R^2^ = 0.94, RSD = 2.88, *p* < 0.01), and SID_Val_ = 151.012 − 2.990TGS (R^2^ = 0.57, RSD = 4.69, *p* < 0.05).

## 4. Discussion

### 4.1. Chemical Composition and AA Profile of Rapeseed Cake

The information of the rest of our samples is shown in Table 1. The majority of the analyzed chemical compositions of the 10 RSC samples in our study were within the range of the tabulated values [4,6,22], indicating the reliability of our obtained data. In this experiment, there was high variability (CV > 10%) in EE, CF, NDF, ADF, and TGS among the 10 RSC samples, indicating significant differences among the RSC samples. The average CP content was slightly higher than the reported values [4,6,22], but lower than some observed values [5,8]. These differences may be due to variations in rapeseed meal varieties and processing techniques. Research indicates that the traits and genetic selection of rapeseed can result in differences in its crude protein content [23]. Additionally, differences in processing conditions directly affect the EE content and other nutrients in RSC [2,24,25,26,27,28,29]. The average GE was similar to the reported values in the Nutrient Requirements of Swine in China (2020) [4] and by Li et al. (2015) [22], but lower than those in the NRC (2012) [6]. The mean CF, ADF, and NDF contents were within the range of tabulated values [4,6,22]. Among them, the NDF of the RSC7 sample was as high as 48.61%, which might be due to the excessive temperature during its thermal processing, resulting in a vigorous Maillard reaction. In the study by Mosenthin et al. [30], the NDF content of rapeseed meal (based on DM) varied between 40.7% and 47.6%, and it was pointed out that the occurrence of the Maillard reaction in rapeseed meal under heating and prolonged heating time would lead to an increase in NDF content. The TGS content of all 10 RSC samples was below 30 μmol/g, aligning with the range in previous studies [31,32]. The Ca and TP contents were also close to the values reported in previous literature [4,6].

Lys, Met, Thr, and Trp are crucial limiting amino acids for pig growth [33,34,35,36,37], which play an indispensable role in pig growth and development [33,34,35,38,39]. The CV values for the Lys and Met content in our RSC samples exceeded 20%, and those for Thr and Trp content were more than 10%, indicating significant differences in the amino acid compositions of our RSC samples. These changes in amino acids may result from different natural conditions and processing methods [30,40,41]. Through multivariate analysis of variance, we found that there was no significant difference (*p* > 0.05) in the overall amino acid composition of the ten samples compared with the NRC (2012) [6] and the Nutrient Requirements of Swine in China (2020) [4]. And they were closely consistent with some reports [4,6,7,22].

### 4.2. SID of AA in Rapeseed Cake

SID was derived based on AID by correcting with basic endogenous losses. One advantage of doing this was that it made the SID values additive [42]. Generally, nitrogen-free diets were used to obtain basic endogenous losses [3]. In Table 6, we compared the basic endogenous losses obtained by previous researchers in growing pigs. The results showed that our basic endogenous losses were basically within or close to the range of the table values. Mathai [18], Espinosa et al. [19], Son et al. [20], and Li et al. [9] all used the nitrogen-free diet method to determine the basic endogenous losses, and the cellulose proportions of their nitrogen-free diets were 3%, 4%, 4%, and 4%, respectively, but the method adopted by Zhang et al. [21] was not described in their report. Studies showed that fiber could seriously affect endogenous losses [43], and the fiber content of the nitrogen-free diet method, under the suggestions of previous researchers, had basically remained within the range of 3% to 4% [10]. The cellulose proportion of our nitrogen-free diet was 4%, which was consistent with the cellulose usage range of previous researchers.

The mean AID and SID values for Lys and Met were lower than those reported in the NRC (2012) [6] (Lys, 70% and 71%; Met, 82% and 83%) and the Nutrient Requirements of Swine in China (2020) [4] (Lys, 66% and 69%; Met, 88% and 89%), which may be related to the fiber components and the processing of the RSC. Several studies have shown that fiber components, such as NDF, can hinder the absorption of other nutrients and increase the endogenous losses of amino acids [10,24,29,44,45]. Elkund et al. [46] found that in the case where the NDF content of the sample (47.6%) was similar to that of our RSC7 sample with the highest NDF content, the SID_Lys_ of the low glucosinolate rapeseed meal obtained was 55%, which was lower than that of the NRC (2012) [6] and the Nutrient Requirements of Swine in China (2020) [4]. 

According to the additional amount of RSC in our diet, the content of NDF contributed by RSC in the diet is approximately between 8.8% and 19.4%. Previous studies [47] have reported that adding wheat bran and soybean hulls to the diet would reduce the AID of dietary AA. Adding 2.5% of wheat bran compared to 0% (dietary NDF level, 14.2% vs. 13.5%) would reduce the AID of AA by 1.4% to 5.2%. Adding 2.5% of soybean hulls compared to 0% (dietary NDF level, 14.8% vs. 13.5%) would reduce the apparent ileal digestibility of amino acids by 4.7% to 10.8%. However, further adding wheat bran or soybean meal at a ratio of 2.5% to 5% or 7.5% had little effect on the AID of AA, and it was pointed out that different types of NDF have different effects on reducing the AID of AA. The inhibitory effect of fibers on the apparent digestibility of amino acids might mainly be due to the cause of higher endogenous amino acid losses, and it was speculated that this endogenous loss has a plateau after increasing [47]. But there were also studies indicating that as the NDF increases, the AID of AA decreases linearly [47,48]. In our study, the predictive equation shows that the SID of lysine has a linear relationship with NDF; the reason for this phenomenon might be that the Maillard reaction caused lysine to combine with the fibrous components, forming an indigestible part. Fiber components could undergo Maillard reactions with AA during the heating process, especially Lys, which was highly susceptible to Maillard reactions with reducing sugars due to its exposed ε-amino group [33]. This reaction leads to the binding of AA to fiber components, making them unavailable for absorption [33]. The lowest SID values or AID of lysine in our two RSC samples were similar to or higher than those of Seneviratne et al. (cold-pressed rapeseed cake: AID_Lys_, 40.8%; SID_Lys_, 41.4%) [45], Almeida et al. (rapeseed meal autoclaved at 130 °C for 45 min: AID_Lys_, 12.9%; SID_Lys_, 20.8%) [49], and Li et al. (2002) (rapeseed cake heated at >130 °C for 25 min, AID_Lys_, 40.6%) [50]. Considering the sample information in our Table 1, it could be seen that in the case of the highest heating temperature and similar time for the RSC2 and RSC7 samples in our study, the digestibility of lysine was also extremely similar. Such a phenomenon indicated that the low digestibility of lysine was the multiple result of endogenous losses, Maillard reactions, and other effects. The lowest AID and SID values of methionine in our study were lower than those of Almeida et al. (rapeseed meal autoclaved at 130 °C for 45 min: AID_Met_, 64%; SID_Met_, 68.5%) [49], Li et al. (2002) (rapeseed cake heated at >130 °C for 25 min: AID_Met_, 83.8%) [50], and Elkund et al. (rapeseed cake with NDF content of 47.6%: SID_Met_, 81%) [46]. The digestibility of most methionine in our samples was higher than or close to the results of Almeida et al. [49]. Considering the sample information of ours, the relationship between the digestibility of methionine in our samples and the processing temperature and time seemed difficult to capture, and the factors causing the low digestibility of methionine might be more complex.

The average AID values of Thr and Trp were similar to those in previous studies [4,6,22,24], but the SID values were slightly higher than the reported values [4,6,22,24,51]. This may be ascribed to a higher level of endogenous losses of Thr and Trp measured in this experiment compared to previous studies [6,32,33,52]. Additionally, the range of SID of proline in this study falls within reported values in the NRC (2012) [6] and the Nutrient Requirements of Swine in China (2020) [4].

### 4.3. Correlation Analysis and Prediction Equations for SID of AA in Rapeseed Cake 

Our study found that the SID _Lys_ had a negative correlation with CF, NDF, ADF, and heating temperature, and the NDF was the best predictor for the prediction equation of SID_Lys_ and SID_CP_. This suggested that fiber components, especially NDF, are critical for the digestion and absorption of AA. Fiber components could undergo Maillard reactions with AA during the heating process, especially Lys, which was highly susceptible to Maillard reactions with reducing sugars due to its exposed ε-amino group [33]. This reaction led to a portion of the protein becoming a component of the NDF matrix and no longer being free during digestion in the small intestine [53]. This also explained why there was a significant negative correlation between heating temperature and SID_Lys_ in our study and why the heating temperature becomes its predictor. In addition, NDF and its lignin fraction had a high-water retention capacity and thereby increased the viscosity of the digesta, which slowed mass transfer and enzymatic reactions, and hence the digestion and absorption of nutrients [10,24,29,44,45,53]. Moreover, the SID of AA and CP was achieved via apparent ileal digestibility coefficients correcting for the ileal basal endogenous losses, and dietary composition, especially fiber components, was responsible for ileal basal endogenous CP and AA losses [9]. While there are no direct findings in previous studies to support our findings, Messad et al. [53] reported that NDF was negatively correlated with SID_Lys_, which was the best predictor for SID_Lys_ and the essential amino acid SID of rapeseed meal in growing pigs. Toghyani et al. [54] found that NDF was negatively correlated with SID_Lys_ and SID_CP_ in canola meal for broiler chickens. NDF was also the single predictor for SID_CP_ from the study on barley fed to pigs [9]. These studies indicated that the nutritional level of rapeseed cake meal could be improved via controlling the fiber contents. 

In this study, the single predictor for SID_Val_ was TGS. Pigs are sensitive to TGS [55]; Wang et al. [56] reported that serum triiodothyronine and tetraiodothyronine concentrations were linearly decreased with the increased contents of double-low rapeseed cake. Spiegel et al. [57] reported TGS induced hypothyroidism, leading to delayed gastric emptying, and reduced intestinal motility and bacterial overgrowth, which affected amino acid absorption [58,59]. However, factors such as fiber fractions might have masked the effect of TGS on SID_AA_ [9,10,27,32,50,51,59], making the influence of TGS difficult to observe in studies. This led to fewer researchers having results similar to us, but a previous study reported that TGS was negatively correlated with SID_Val_ in RSC for laying hens [60]. Unfortunately, there is no literature specifically explained the effect of TGS on amino acid digestion and absorption and its mechanism of influence. More efforts and works are needed to do so in the future.

### 4.4. Validate the Prediction Equations for SID_CP_ and SID_AA_ of Rapeseed Cake Based on Database

The predicted SID_CP_ and SID_Lys_ values for RSC using the best prediction equations were 77.66% and 70.90%, respectively, which were similar to the reported values in the NRC (2012) [6] (75% and 71%). Since the NRC (2012) [6] lacks the TGS value, we used the parameters from Seneviratne [61] and Li et al. [22]. The predicted values of SID_Val_ were 78.44% and 74.83%, respectively, which were higher than or similar to the reported values [22,61] of 70.5% and 76.93%.

## 5. Conclusions

The source and processing factors significantly affected the chemical composition and AA profile of RSC in the Yangtze River Basin and its adjacent areas, resulting in great variations in the digestibility of AA for pigs. NDF, TGS, and heating temperature might be the predictors for the SID of AA. 

## Figures and Tables

**Table 1:** Sources of rapeseed cake.

Sample Numbers	Colors *	Source of Variety	Heating Temperature and Time	Sources	Storage Conditions
RSC1	brown	*Brassica napus* L.	130 ± 10 °C, 30 min	Liupanshui city, Guizhou	Normal temperature, <3 months
RSC2	reddish brown	*Brassica napus* L.	165 ± 15 °C, 25 min	Neijiang city, Sichuang	Normal temperature, <3 months
RSC3	yellowish green	*Brassica napus* L.	115 ± 10 °C, 30 min	Huainan city, Anhui	Normal temperature, <3 months
RSC4	greener	*Brassica napus* L.	40 ± 20 °C, 60~120 min	Hanzhong city, Shaanxi	Normal temperature, <3 months
RSC5	yellowish green	*Brassica napus* L.	95 ± 10 °C, 25 min	Xuchang city, Henan	Normal temperature, <3 months
RSC6	yellowish green	*Brassica napus* L.	115 ± 10 °C, 30 min	Xiantao city, Hubei	Normal temperature, <3 months
RSC7	reddish brown	*Brassica napus* L.	165 ± 15 °C, 25 min	Huaihua city, Hunan	Normal temperature, <3 months
RSC8	brown	*Brassica napus* L.	130 ± 10 °C, 30 min	Chongqing Municipality	Normal temperature, <3 months
RSC9	brown	*Brassica napus* L.	130 ± 10 °C, 30 min	Xiangxiang city, Hunan	Normal temperature, <3 months
RSC10	yellowish brown	*Brassica napus* L.	95 ± 10 °C, 60 min	Hanchuan city, Hubei	Normal temperature, <3 months

RSC, rapeseed cake; * The color information was derived from the color of the entire powder after comminution.

**Table 2:** Ingredient composition of experimental diet and nitrogen-free diet (air-dry basis, %).

Items	Experimental Diet	Nitrogen-Free Diet
Maize starch	42.90	78.90
Rapeseed cake	40.00	
Soybean oil	3.00	3.00
Cellulose acetate		4.00
Sucrose	10.00	10.00
Limestone	0.5	0.5
Calcium phosphite (Ca(H_2_PO_4_)_2_)	1.9	1.9
Titanium dioxide (TiO_2_)	0.3	0.3
Sodium chloride (NaCl)	0.4	0.4
Potassium carbonate (K_2_CO_3_)	0.4	0.4
Magnesium oxide (MgO)	0.1	0.1
Vitamin and mineral premix	0.5	0.5
Total	100.00	100.00

The vitamin and mineral premix provided the following per kg of diets: VA 4200 IU, VD_3_ 400 IU, VE 36 IU, VK_3_ 1.2 mg, VB_12_ 23 ug, VB_2_ 5.63 mg, VB_5_ 20.5 mg, VB_3_ 28 mg, choline chloride 1.00 g, folic acid 0.8 mg, VB_1_ 3.4 mg, VB_6_ 2.7 mg, VH 0.18 mg, Mn (as manganese sulfate) 40.0 mg, Fe (as ferrous sulfate) 70.0 mg, Zn (as copper sulfate) 70 mg, I (as potassium iodide) 0.3 mg, Se (as sodium selenite) 0.3 mg.

**Table 3:** Analyzed chemical composition, physical characteristics of 10 rapeseed cake samples (air-dry basis, %).

Items	Rapeseed Cake Number										Mean	CV (%)
	RSC1	RSC2	RSC3	RSC4	RSC5	RSC6	RSC7	RSC8	RSC9	RSC10		
DM	93.02	87.95	90	91.67	92.36	91.57	94.68	92.06	91.08	91.85	91.62	1.94
GE, MJ/kg	19.08	19.07	18.6	19.47	18.45	20.11	20.84	18.94	18.72	19.18	19.25	3.8
CP	43.61	38.66	38.48	39.33	42.78	35.15	38.37	41.73	36.67	36.76	39.15	7.07
EE	7.96	5.81	7.98	7.76	6.46	11.76	9.73	7.54	7.92	8.13	8.11	32.79
Ash	7.07	6.62	6.11	5.99	7.75	6.42	7.09	7.02	6.78	6.89	6.77	7.65
CF	8.26	10.25	9.34	8.81	8.4	9.72	11.32	8.55	9.29	9.5	9.34	10.01
NDF	30.87	44.25	24.19	23.27	21.89	26.61	48.61	27.77	29.3	25.12	30.19	29.94
ADF	15.83	22.25	16.89	16.28	14.71	15.91	22.87	13.13	19.59	19.39	17.68	18.19
Ca	0.58	0.58	0.5	0.57	0.62	0.56	0.59	0.57	0.62	0.6	0.58	5.92
TP	1.25	1.08	0.92	1.19	1.19	1.05	1.3	1.27	1.17	1.18	1.16	9.67
TGS/(μmol/g)	21.84	22.81	23.82	20.27	21.39	22.97	25.02	20.25	20.86	24.27	22.35	15.19
Essential amino acids, %												
Arginine	2.86	2.03	2.54	2.55	2.48	2.18	2.32	2.45	2.45	1.74	2.36	13.18
Histidine	1.78	1.52	1.65	1.59	1.58	1.60	1.56	1.60	1.63	1.13	1.56	10.79
Isoleucine	2.63	1.54	1.58	2.14	1.56	2.06	2.07	1.99	2.02	1.16	1.87	22.13
Leucine	2.29	2.29	2.39	2.40	2.34	2.38	2.24	2.24	2.33	1.98	2.29	5.35
Lysine	2.20	1.22	1.91	2.08	2.14	2.04	1.23	1.42	1.73	2.08	1.80	21.13
Methionine	0.90	0.73	0.77	0.74	0.76	0.95	0.82	1.29	0.71	0.82	0.85	20.30
Phenylalanine	1.96	1.61	1.69	1.21	1.66	1.25	1.14	1.16	1.10	1.48	1.43	20.55
Threonine	1.97	1.65	1.64	1.60	1.73	1.66	1.45	1.58	1.59	1.20	1.61	12.14
Tryptophan	0.36	0.30	0.25	0.28	0.29	0.29	0.37	0.36	0.27	0.33	0.31	13.41
Valine	2.68	2.25	2.34	2.14	2.31	2.28	1.81	2.84	2.32	1.88	2.29	13.72
Non-essential amino acids, %												
Alanine	2.12	1.64	1.66	1.69	1.62	1.76	1.57	1.60	1.60	1.28	1.65	12.57
Aspartate	3.17	2.39	2.67	2.49	2.49	2.53	2.36	2.47	2.48	1.99	2.50	11.73
Cystine	1.14	1.05	1.11	1.03	1.23	1.69	1.10	1.14	1.13	1.01	1.16	16.67
Glutamine	8.55	5.83	6.70	6.40	6.57	6.11	5.94	5.92	6.22	5.40	6.36	13.44
Glycine	2.25	1.65	1.71	1.70	1.69	1.74	1.61	1.63	1.67	1.45	1.71	12.06
Proline	2.28	1.83	1.87	3.91	2.07	3.17	3.44	3.71	2.24	1.95	2.65	30.92
Serine	2.23	1.55	1.63	1.60	1.62	1.63	1.42	1.61	1.63	2.31	1.72	17.15
Tyrosine	1.84	1.29	1.43	1.30	1.41	1.38	1.27	1.35	1.33	0.74	1.33	20.00

DM, dry matter; GE, gross energy; CP, crude protein; EE, ether extract; Ash, crude ash; CF, crude fiber; NDF, neutral detergent fiber; ADF, acid detergent fiber; Ca, calcium; TP, total phosphorus; TGS, total glucosinolate; CV, coefficient of variation.

**Table 4:** Analyzed chemical composition of experiment diets (air-dry basis, %).

	Rapeseed Cake Diet										Mean	CV (%)	N-Free Diet
Items	1	2	3	4	5	6	7	8	9	10			
DM,%	90.85	90.20	90.61	90.36	92.34	91.11	92.89	92.46	90.89	91.30	91.30	1.03	90.28
CP,%	17.71	12.94	14.04	14.19	13.88	14.18	12.82	14.21	13.52	11.59	13.91	11.33	1.45
Essential amino acids, %													
Arginine	1.15	0.80	1.00	0.99	0.97	0.84	0.89	0.96	0.96	0.67	0.92	14.00	-
Histidine	0.72	0.60	0.65	0.62	0.62	0.61	0.59	0.62	0.64	0.44	0.61	11.52	-
Isoleucine	1.05	0.61	0.62	0.83	0.61	0.79	0.79	0.78	0.78	0.45	0.73	22.69	-
Leucine	0.92	0.90	0.94	0.93	0.92	0.91	0.86	0.88	0.91	0.77	0.89	5.68	-
Lysine	0.89	0.48	0.75	0.81	0.84	0.78	0.47	0.56	0.67	0.80	0.70	21.43	-
Methionine	0.36	0.29	0.30	0.29	0.30	0.36	0.32	0.51	0.28	0.32	0.33	20.46	-
Phenylalanine	0.78	0.63	0.67	0.47	0.65	0.48	0.44	0.45	0.43	0.57	0.56	21.60	-
Threonine	0.79	0.65	0.65	0.63	0.68	0.64	0.56	0.62	0.62	0.46	0.63	13.13	-
Tryptophan	0.15	0.12	0.10	0.11	0.11	0.11	0.14	0.14	0.10	0.13	0.12	13.57	-
Valine	1.08	0.89	0.92	0.83	0.90	0.88	0.69	1.11	0.90	0.72	0.89	14.72	-
Non-essential amino acids, %													-
Alanine	0.86	0.65	0.65	0.66	0.63	0.68	0.60	0.63	0.62	0.49	0.65	14.02	-
Aspartate	1.28	0.94	1.04	0.97	0.97	0.97	0.90	0.96	0.96	0.76	0.98	13.05	-
Cystine	0.46	0.41	0.43	0.40	0.48	0.65	0.42	0.45	0.44	0.39	0.45	16.38	0.08
Glutamine	3.40	2.30	2.64	2.48	2.59	2.36	2.26	2.31	2.41	2.08	2.48	14.48	-
Glycine	0.91	0.65	0.68	0.66	0.67	0.67	0.61	0.64	0.65	0.56	0.67	13.47	-
Proline	0.91	0.72	0.74	1.51	0.82	1.22	1.32	1.44	0.87	0.75	1.03	30.20	0.51
Serine	0.89	0.61	0.64	0.62	0.63	0.63	0.54	0.63	0.63	0.89	0.67	17.53	-
Tyrosine	0.74	0.51	0.56	0.50	0.55	0.53	0.48	0.53	0.52	0.29	0.52	21.18	-

DM, dry matter; CP, crude protein; CV, coefficient of variation.

**Table 5:** Apparent ileal digestibility (AID) of crude protein (CP) and amino acids (AA) in rapeseed cake fed to growing-finishing pigs (%).

	Rapeseed Cake										Mean	SEM	*p*-Value
Items	RSC1	RSC2	RSC3	RSC4	RSC5	RSC6	RSC7	RSC8	RSC9	RSC10			
CP, %	70.29 ^a^	52.19 ^de^	62.36 ^abc^	63.38 ^abc^	66.29 ^ab^	66.09 ^ab^	46.54 ^e^	54.76 ^cde^	60.46 ^bcd^	54.81 ^cde^	59.72	1.29	<0.01
Essential amino acids, %													
Arginine	75.56 ^ab^	70.61 ^ab^	80.29 ^a^	77.19 ^ab^	75.38 ^ab^	78.09 ^ab^	75.59 ^ab^	73.39 ^ab^	79.82 ^ab^	68.94 ^b^	75.49	1.07	0.28
Histidine	73.22 ^cd^	72.62 ^cd^	76.80 ^bc^	68.01 ^d^	67.27 ^d^	73.71 ^cd^	81.92 ^ab^	86.27 ^a^	88.41 ^a^	83.20 ^ab^	77.14	1.26	<0.01
Isoleucine	81.20 ^ab^	71.92 ^d^	72.45 ^cd^	80.09 ^ab^	76.50 ^bcd^	84.81 ^a^	78.38 ^abc^	78.73 ^ab^	81.65 ^ab^	66.13 ^e^	77.19	0.91	<0.01
Leucine	72.30 ^ab^	73.35 ^ab^	76.54 ^ab^	75.59 ^ab^	79.96 ^a^	81.19 ^a^	77.42 ^a^	60.59 ^bc^	51.72 ^c^	53.25 ^c^	70.19	2.04	<0.01
Lysine	64.34 ^a^	41.12 ^b^	65.21 ^a^	67.90 ^a^	71.61 ^a^	68.56 ^a^	39.94 ^b^	64.70 ^a^	64.12 ^a^	72.23 ^a^	61.97	2.16	<0.01
Methionine	64.76 ^b^	53.52 ^bc^	46.08 ^c^	58.70 ^bc^	49.67 ^c^	52.17 ^c^	83.92 ^a^	81.18 ^a^	81.99 ^a^	81.05 ^a^	65.31	2.39	<0.01
Phenylalanine	76.87 ^b^	74.26 ^b^	76.44 ^b^	67.30 ^c^	76.58 ^b^	75.29 ^a^	83.22 ^a^	88.51 ^a^	88.89 ^a^	85.34 ^a^	79.27	1.08	<0.01
Threonine	68.20 ^ab^	64.24 ^ab^	66.55 ^ab^	62.81 ^b^	65.36 ^ab^	71.63 ^ab^	66.37 ^ab^	68.24 ^ab^	75.86 ^a^	69.58 ^ab^	67.88	1.16	0.37
Tryptophan	84.12 ^a^	80.20 ^ab^	73.75 ^c^	78.11 ^abc^	80.59 ^ab^	81.69 ^ab^	81.40 ^ab^	83.36 ^a^	75.91 ^bc^	79.98 ^ab^	79.91	0.68	<0.01
Valine	81.62 ^a^	77.76 ^ab^	77.99 ^ab^	80.57 ^ab^	83.36 ^a^	79.91 ^ab^	68.88 ^bc^	82.89 ^a^	82.55 ^a^	59.86 ^c^	77.54	1.48	<0.01
Non-essential amino acids, %													
Alanine	73.80 ^ab^	68.54 ^ab^	70.70 ^ab^	69.53 ^ab^	73.43 ^ab^	74.34 ^a^	64.28 ^b^	68.89 ^ab^	75.35 ^a^	67.39 ^ab^	70.62	0.96	0.20
Aspartate	73.74	67.17	71.78	69.05	72.67	73.33	66.47	67.98	73.88	66.22	70.23	0.88	0.21
Cystine	67.90 ^bc^	66.84 ^bc^	71.11 ^abc^	64.24 ^c^	76.15 ^abc^	60.58 ^c^	80.28 ^ab^	83.27 ^a^	82.67 ^a^	69.21 ^bc^	72.23	1.56	<0.01
Glutamine	86.46 ^ab^	80.33 ^bc^	83.02 ^abc^	81.26 ^bc^	83.79 ^abc^	84.37 ^abc^	77.10 ^c^	82.10 ^bc^	89.62 ^a^	83.73 ^abc^	83.18	0.79	<0.05
Glycine	74.65 ^a^	63.50 ^ab^	66.55 ^ab^	62.44 ^ab^	60.50 ^ab^	62.30 ^ab^	32.28 ^c^	35.01 ^c^	56.10 ^b^	57.49 ^b^	57.08	2.17	<0.01
Proline	55.06 ^a^	47.90 ^ab^	52.08 ^ab^	63.70 ^a^	24.90 ^b^	50.26 ^ab^	44.90 ^ab^	63.77 ^a^	61.72 ^a^	46.77 ^ab^	51.11	2.83	0.33
Serine	78.99 ^abc^	69.58 ^cd^	75.54 ^abc^	71.68 ^bcd^	75.14 ^abcd^	69.31 ^cd^	65.26 ^d^	74.37 ^bcd^	80.84 ^ab^	84.47 ^a^	74.52	1.16	<0.01
Tyrosine	79.09 ^a^	73.49 ^ab^	75.87 ^ab^	75.02 ^ab^	78.86 ^a^	79.40 ^a^	74.98 ^ab^	70.97 ^ab^	76.49 ^ab^	67.82 ^b^	75.20	0.92	0.09

CP, crude protein; ^a,b,c,d,e^ Means that values in the same row with no letter or the same letter are not different at *p* < 0.05.

**Table 6:** Analysis of basal endogenous loss of crude protein and amino acids (g/kg DM intake).

IAAend	Mathai [18]	Espinosa et al. [19]	Son et al. [20]	Zhang et al. [21]	Li et al. [9]	Min	Max	Mean	Our Study
Arg	1.01	0.89	2.14	0.14	0.43	0.14	2.14	0.92	1.96
His	0.31	0.19	0.25	0.11	0.09	0.09	0.31	0.19	0.23
Ile	0.57	0.32	0.37	0.18	0.28	0.18	0.57	0.34	0.56
Leu	0.9	0.48	0.6	0.21	0.38	0.21	0.90	0.51	0.43
Lys	0.91	0.62	0.54	0.09	0.36	0.09	0.91	0.50	0.08
Met	0.16	0.08	0.11	0.04	0.08	0.04	0.16	0.09	0.15
Met + Cys	-	-	0.32	-	-	0.32	0.32	0.32	-
Phe	0.55	0.29	0.36	0.28	0.21	0.21	0.55	0.34	0.48
Thr	0.86	0.55	0.67	0.41	0.48	0.41	0.86	0.59	0.8
Trp	0.2	0.09	0.19	-	0.1	0.09	0.20	0.15	0.19
Val	0.93	0.41	0.53	0.51	0.35	0.35	0.93	0.55	0.6
Ala	1.01	0.68	1.43	0.26	0.61	0.26	1.43	0.80	1.01
Asp	1.32	0.78	1.09	0.49	0.8	0.49	1.32	0.90	0.77
Cys	0.29	0.2	0.2	0.14	0.07	0.07	0.29	0.18	0.04
Glu	1.58	0.94	1.3	0.63	1.06	0.63	1.58	1.10	1.03
Gly	2.57	1.94	3.77	0.61	1	0.61	3.77	1.98	1.02
Pro	-	-	20.1	2.47	1.26	1.26	20.10	7.94	6.39
Ser	0.8	0.55	0.84	0.35	0.49	0.35	0.84	0.61	0.64
Tyr	0.45	0.25	-	0.38	0.14	0.14	0.45	0.31	0.63
CP	-	20.27	36.3	-	10.71	10.71	36.30	22.43	21.24

**Table 7:** Standardized ileal digestibility (SID) of crude protein (CP) and amino acids (AA) in rapeseed cake fed to growing-finishing pigs (%).

	Rapeseed Cake										Mean	SEM	*p*-Value
Items	RSC1	RSC2	RSC3	RSC4	RSC5	RSC6	RSC7	RSC8	RSC9	RSC10			
CP, %	81.19 ^a^	67.00 ^de^	76.07 ^abc^	76.9 ^abc^	80.42 ^ab^	79.73 ^ab^	61.92 ^e^	68.59 ^cde^	74.74 ^abcd^	71.54 ^bcd^	73.81	1.18	<0.001
Essential amino acids, %													
Arginine	91.04	92.55	98.09	95.04	93.9	99.27	96.06	92.23	98.4	95.48	95.21	1.02	0.72
Histidine	76.17 ^cd^	76.14 ^cd^	80.05 ^bc^	71.42 ^d^	70.73 ^d^	77.16 ^cd^	85.56 ^ab^	89.71 ^a^	91.73 ^a^	88.07 ^ab^	80.68	1.27	<0.01
Isoleucine	86.05 ^ab^	80.22 ^bc^	80.72 ^bc^	86.21 ^ab^	85.04 ^ab^	91.28 ^a^	85 ^ab^	85.44 ^ab^	88.19 ^a^	77.55 ^c^	84.57	0.77	<0.01
Leucine	76.53 ^ab^	77.65 ^ab^	80.67 ^ab^	79.75 ^ab^	84.27 ^a^	85.48 ^a^	82.06 ^a^	65.11 ^bc^	56.01 ^c^	58.35 ^c^	74.59	2.02	<0.01
Lysine	65.13 ^a^	42.56 ^b^	66.14 ^a^	68.75 ^a^	72.45 ^a^	69.46 ^a^	41.45 ^b^	65.98 ^a^	65.16 ^a^	73.11 ^a^	63.02	2.14	<0.01
Methionine	68.50 ^b^	58.17 ^bc^	50.56 ^c^	63.35 ^bc^	54.25 ^c^	55.89 ^bc^	88.28 ^a^	83.89 ^a^	86.86 ^a^	85.35 ^a^	69.51	2.37	<0.01
Phenylalanine	82.48 ^bc^	81.12 ^bc^	82.99 ^bc^	76.56 ^c^	83.4 ^bc^	84.35 ^b^	93.44 ^a^	98.38 ^a^	99.03 ^a^	93.04 ^a^	87.48	1.17	<0.01
Threonine	77.48 ^ab^	75.38 ^b^	77.77 ^ab^	74.42 ^b^	76.23 ^ab^	83.11 ^ab^	79.75 ^ab^	80.25 ^ab^	87.66 ^a^	85.42 ^ab^	79.75	1.19	0.17
Tryptophan	96.07	94.77	91.41	93.7	96.26	97.76	93.97	96.05	92.48	93.84	94.63	0.60	0.43
Valine	86.98 ^a^	84.24 ^a^	84.28 ^a^	87.48 ^a^	89.87 ^a^	86.50 ^a^	77.49 ^ab^	88.21 ^a^	88.97 ^a^	67.88 ^c^	84.19	1.42	<0.01
Non-essential amino acids, %													
Alanine	84.45 ^ab^	82.60 ^ab^	84.78 ^ab^	83.38 ^ab^	88.14 ^ab^	87.94 ^ab^	79.83 ^b^	83.77 ^ab^	90.09 ^a^	86.10 ^ab^	85.11	0.94	0.40
Aspartate	79.25	74.56	78.49	76.27	80.00	80.57	74.43	75.40	81.17	75.46	77.56	0.85	0.47
Cystine	76.69 ^bc^	76.51 ^bc^	80.32 ^abc^	74.20 ^c^	79.87 ^abc^	66.76 ^c^	90.02 ^ab^	92.42 ^a^	91.76 ^a^	79.52 ^abc^	80.80	1.58	<0.01
Glutamine	89.23 ^ab^	84.38 ^bc^	86.56 ^abc^	85.03 ^abc^	87.48 ^abc^	88.36 ^abc^	81.35 ^c^	86.24 ^abc^	93.52 ^a^	88.26 ^abc^	87.04	0.78	<0.05
Glycine	84.86 ^a^	77.54 ^cd^	80.22 ^a^	76.36 ^a^	74.57 ^a^	76.12 ^a^	47.70 ^b^	49.84 ^b^	70.30 ^a^	74.11 ^a^	71.16	2.09	<0.01
Proline	118.68 ^ab^	127.87 ^a^	130.46 ^a^	101.85 ^abc^	95.63 ^bc^	97.43 ^bc^	88.76 ^b^	103.74 ^abc^	128.22 ^a^	123.64 ^ab^	111.63	3.38	<0.01
Serine	85.58 ^ab^	79.05 ^b^	84.68 ^ab^	81.01 ^ab^	84.5 ^ab^	78.68 ^b^	76.25 ^b^	83.83 ^ab^	90.07 ^a^	91.06 ^a^	83.47	1.08	0.021
Tyrosine	86.76	84.49	86.09	86.26	89.38	90.12	87.13	81.96	87.51	87.92	86.76	0.84	0.70

CP, crude protein; Values for SID were calculated by correcting the AID values with the basal endogenous losses (IAA_end_). IAA_end_ (g/kg DM intake) averaged as CP, 21.24; Arg, 1.96; His, 0.23; Ile, 0.56; Leu, 0.43; Lys, 0.08; Met, 0.15; Phe, 0.48; Thr, 0.80; Trp, 0.19; Val, 0.6; Ala, 1.01; Asp, 0.77; Cys, 0.04; Glu,1.03; Gly, 1.02; Pro, 6.39; Ser, 0.64; Tyr, 0.63. ^a,b,c,d,e^ Means that values in the same row with no letter or the same letter are not different at *p* < 0.05.

**Table 8:** Stepwise regression equations for SID of CP, Lys, Met, Arg, and Val based upon the chemical characteristics of the 10 rapeseed cake samples (air-dry basis, %).

Items	Prediction Equations	RSD	R^2^	*p*-Value
SID_CP_	SID_CP_ = 90.124 − 0.540NDF	4.39	0.58	<0.05
SID_Lys_	SID_Lys_ = 100.107 − 1.229NDF	2.88	0.94	<0.01
SID_Lys_	SID_Lys_ = 90.662 − 0.234HT	8.04	0.51	<0.05
SID_Val_	SID_Val_ = 151.012 − 2.990TGS	4.69	0.57	<0.05

SID, standardized ileal digestibility; CP, crude protein; NDF, neutral detergent fiber; HT, heating temperature; TGS, total glucosinolate; Lys, Lysine; Val, valine; R^2^, R-square; RSD, relative standard deviation. *p* < 0.05 means significant difference; *p* < 0.01 means extremely significant difference.

## Data Availability

The original contributions presented in the study are included in the article, further inquiries can be directed to the corresponding author.
